# New Health Reform Planning Tool for State and Local Health Departments

**Published:** 2013-07-05

The Georgia Health Policy Center has released “Leading Through Health System Change: A Public Health Opportunity,” a new tool to help public health organizations adapt to the changing health-care environment. The tool’s interactive website can be used to examine the basics of health reform, apply adaptive thinking to questions of health system change, and create a simple implementation plan to increase opportunities for improving population health. The tool was created through a cooperative agreement with CDC and the National Network of Public Health Institutes. The tool is available at http://www.metacat.net/metacat/app/ghpc.

